# Comparison of Outcomes of Haploidentical Peripheral Blood Stem Cell Transplantation with Post-Transplant Cyclophosphamide in Older Versus Younger Patients

**DOI:** 10.3390/cancers17020310

**Published:** 2025-01-19

**Authors:** Giacomo Adoncecchi, Ambuj Kumar, Krishnakar Mogili, Rawan Faramand, Hien Liu, Farhad Khimani, Asmita Mishra, Michael Nieder, Taiga Nishihori, Doris Hansen, Michael Jain, Aleksandr Lazaryan, Lia Perez, Joseph Pidala, Frederick Locke, Claudio Anasetti, Nelli Bejanyan, Hany Elmariah

**Affiliations:** 1 Department of Bone Marrow Transplant and Cellular Immunotherapy, H. Lee Moffitt Cancer Center, Tampa, FL 33612, USA; gadoncecchi@health.ucsd.edu (G.A.); doris.hansen@moffitt.org (D.H.); frederick.locke@moffitt.org (F.L.);; 2 Department of Internal Medicine, Morsani College of Medicine, University of South Florida, Tampa, FL 33612, USA; akumar6@usf.edu

**Keywords:** haploidentical, post-transplant cyclophosphamide, elderly, transplant

## Abstract

Background: Previous studies have shown that allogeneic peripheral blood stem cell transplantation (PBSCT) from an HLA haploidentical (haplo) donor followed by graft-versus-host disease (GVHD) prophylaxis with post-transplant cyclophosphamide (PTCy) results in lower relapse rates and improved DFS when compared to haplo bone marrow transplant (BMT) with PTCy. However, PBSCT leads to higher rates of GVHD. It is unknown whether the benefits of haplo PBSCT may be nullified in older patients (>60 years) by a higher susceptibility to GVHD and transplant related toxicity. Thus, we sought to determine if older patients receiving haplo PBSCT with PTCy experience significantly worse outcomes than younger patients. Methods: We evaluated 121 adult patients with hematologic malignancies treated at the Moffitt Cancer Center with allogeneic haplo PBSCT followed by PTCy and compared outcomes of patients ≥60 years (n = 55) versus patients <60 years (n = 66). Results: The cumulative incidence of non-relapse mortality (NRM) from the competing risk regression analysis was worse for the older patient group (SHR = 4.05, 95% CI: 1.43–11.47, *p* = 0.008). However, there was no significant difference between groups in graft-versus-host disease (GVHD), relapse, disease-free survival (DFS), or overall survival (OS). Instead, hematopoietic comorbidity index (HCT-CI) ≥ 3 was associated with worse DFS (HR = 1.87, 95% CI: 1.04–3.34, *p* = 0.035) and OS (HR = 1.98, 95% CI: 1.03–3.84, *p*-value = 0.042). Subgroup analysis of patients ≥60 years showed a trend toward improved 2-year OS with fludarabine/cyclophosphamide/total body irradiation (Flu/Cy/TBI) versus fludarabine/busulfan: 71% versus 53% (HR = 0.47, *p* = 0.121). In patients over 70 years (n = 14), NRM was 8% and OS was 76% at 1 year. Conclusion: Given similar OS and DFS between patients aged >60 years and those <60, haplo PBSCT with PTCy appears to be an appropriate transplant platform for older patients.

## Simple Summary

Post-transplant cyclophosphamide (PTCy) for prevention of graft-versus-host disease has allowed for the use of HLA haploidentical donor peripheral blood stem cell transplantation (PBSCT) with outcomes that are similar to historical outcomes with HLA-matched donors. However, data evaluating the safety of this platform in older patients are limited. In this retrospective analysis of patients receiving haploidentical PBSCT with PTCy, patients over 60 years tolerated the regimen, with survival that was similar to a younger control group. Haploidentical PBSCT with PTCy appears to be acceptably safe for use in older patients.

## 1. Introduction

For many hematologic malignancies, the only currently curative treatment regimen is allogeneic hematopoietic cell transplant (HCT). Historically, this treatment was limited only to patients with a human leukocyte antigen (HLA)-matched donor due to excess graft-versus-host disease (GVHD) and non-relapse mortality (NRM) when using HLA-mismatched donors. However, only a minority of patients will have a matched related donor, and the likelihood of finding a matched unrelated donor varies from 10 to 80% depending on the patient’s ethnicity [1]. Including haploidentical-related donors in search algorithms increases the likelihood of finding a suitable donor to nearly 100% [2].

Post-transplant cyclophosphamide (PTCy) has proven to be an effective GVHD prophylaxis platform, allowing for favorable outcomes after HCT even when using HLA-mismatched donors [3,4]. Multiple studies have shown that HLA haploidentical (haplo) donor HCT with PTCy results in rates of GVHD, NRM, and survival that approximate historical outcomes with matched donors [3,5,6,7,8]. Indeed, in some reports, rates of NRM have fallen below 10% when combining a low-intensity conditioning regimen with haplo HCT and PTCy [9,10]. However, some studies have shown that relapse rates may be higher with this GVHD prevention platform, especially in the setting of diseases at high risk for relapse [5,11,12].

The relatively low toxicity profile of this protocol makes it an appealing choice for older patients, though data supporting this practice are limited. Kasamon et al. demonstrated that older patients undergoing haplo bone marrow transplants (BMTs) with PTCy had comparable survival outcomes to younger patients [13]. Notably, relapse was the primary driver of mortality. While the haplo HCT with PTCy platform was developed with bone marrow grafts, many centers preferentially use mobilized peripheral blood stem cell transplants (PBSCTs). In the setting of unrelated donor transplants, Anasetti et al. showed that PBSCT leads to improved engraftment, higher chronic GVHD, and similar survival outcomes compared to bone marrow transplantation in patients with leukemia and lymphoma [14]. In the setting of haploidentical donor HCT with PTCy, PBSCT does appear to yield higher rates of GVHD, but retrospective data have also suggested an improvement in disease-free survival (DFS) with PBSCT, attributable to a reduction in relapse rates without an increase in NRM [15,16]. Thus, haplo PBSCT with PTCy yields higher GVHD rates, but perhaps improved disease control.

While DFS following PBSCT with PTCy is encouraging in the general population, there is a risk that the higher GVHD rates may have a more negative impact on older patients, leading to high NRM and lower DFS in this specific patient population. In addition to baseline frailty, older age is a baseline risk factor for GVHD after transplant, which could limit the feasibility of using PBSC grafts with a haplo with PTCy platform [17,18]. Further, haplo PBSCT with PTCy is correlated with toxicities such as cytokine-release syndrome, viral reactivation, cardiac toxicity, and hemorrhagic cystitis, which could counteract the benefits of the platform in an older patient population [19,20,21,22,23]. Thus, assessment of the outcomes of haplo PBSCT with PTCy in older patients is needed. In this study, we evaluate the safety and efficacy of haplo PBSCT with PTCy in older patients, using outcomes in younger patients as a reference group.

## 2. Methods

### 2.1. Study Population

Data were obtained from the Moffitt Blood and Marrow Transplant Research and Analysis Information Network (BRAIN) database, as well as manual chart review. This study was approved by the Advarra Institutional Review Board (#00000971) at the Moffitt Cancer Center.

This study included 121 consecutive adult patients treated at the Moffitt Cancer Center with allogeneic T-cell replete haploidentical donor PBSCT followed by PTCy-based GVHD prophylaxis for a hematologic malignancy between 11 December 2014 and 12 October 2019. The patients were separated into two groups: one comprised patients who received their transplant when they were <60 years, and the other included patients who received their transplant when they were ≥60 years based on historical precedent and the median age of the cohort [11,12]. Both reduced intensity conditioning (RIC) and myeloablative conditioning (MAC) were included in the study. Specific myeloablative regimens included fludarabine/busulfan (Flu/Bu) or Etoposide/Total Body Irradiation (TBI, >1000 cGy). Reduced intensity regimens were fludarabine/melphalan (Flu/Mel) or flu/cyclophosphamide (Cy)/TBI (200 cGy) [24,25]. For all patients, PTCy was given at a dose of 50 mg/kg/day on days +3 and +4 after HCT. PTCy was used in combination with tacrolimus/mycophenolate mofetil (N = 58), sirolimus/mycophenolate mofetil (N = 63), or sirolimus alone (N = 1). The desired CD34+ cell dose was 5 × 10^6^ CD34+ cells/kg.

### 2.2. Definitions and Endpoints

The primary endpoint of the study was the overall survival (OS) based on the recipient’s age, defined as the time from transplant until the time of death or last follow-up. DFS was defined as time to death or relapse, while non-relapse mortality (NRM) was defined as death in the absence of prior relapse. OS, NRM, DFS, and chronic GVHD were all calculated at 2 years post-transplant, while acute GVHD was measured at day 100. Acute GVHD was graded based on the Keystone Criteria, and determination of chronic GVHD severity was based on the National Institutes of Health criteria [26,27]. Neutrophil engraftment was defined as the first of three consecutive days with an absolute neutrophil count ≥0.5 × 10^9^/L. Platelet engraftment was defined as a platelet count ≥20 × 10^9^/L without requiring a platelet transfusion for at least 7 days prior. The hematopoietic cell transplant comorbidity index (HCT-CI) and disease risk index (DRI) were scored as previously described [28,29,30].

### 2.3. Statistical Analysis

Patient and clinical characteristics were summarized as mean/median, along with SD/IQR, as applicable, for continuous variables and as proportions for categorical variables. The difference in continuous variables across compared groups was assessed using the Mann–Whitney U test and using the Chi-square/Fisher exact test for categorical variables. The crude and adjusted association between categorical dependent outcomes and age group was assessed using the binary logistic regression and summarized as OR, along with 95% confidence intervals (CIs). The unadjusted and adjusted cumulative incidence accounting for competing risks of time-to-event outcomes across compared groups was assessed using the Fine–Gray model and summarized as sub-distribution hazard ratio (SHR), along with 95% CI. The crude and adjusted association between time-to-event outcomes without competing risks and age group was assessed using the Cox proportional hazards model and summarized as HR, along with 95% CI. The statistical significance was set at 0.05 for all comparisons. All analyses were performed using the STATA ver. 18.5 statistical analysis package.

## 3. Results

### 3.1. Baseline Characteristics

Overall, this analysis included 121 patients. The median age for the older group was 66 years (IQR 5.68), and for the younger group, it was 42 years (IQR 19). The median follow-up time for the entire cohort was 11 months (IQR 19) and was 17 months for surviving patients (range: IQR 20). The median CD34+ cell dose was 7.9 × 10^6^ CD34+ cells/kg (IQR 9 × 10^6^ CD34+ cells/kg). The older age group was more likely to have older donors (median donor age of 33, IQR 21 vs. 41, IQR 13; *p* = 0.006). The older age group was more likely to receive a reduced intensity conditioning regimen compared to the younger age group (*p* < 0.001), and it included a higher proportion of myeloid malignancies. Baseline characteristics were otherwise similar between the groups, as shown in Table 1.

### 3.2. Hematopoietic Engraftment

By day 30, the rate of neutrophil engraftment (>500/μL) was similar between the younger and older groups (98% versus 93%, *p* = 0.263). However, the younger group achieved a faster median time to neutrophil engraftment than the older group (16 versus 21 days, *p* < 0.001). By day 90, the rate of platelet engraftment (>20,000/μL) was higher in the younger patients (92% versus 76%, *p* = 0.032). Similarly, the median time to platelet engraftment was faster in the younger group (28 days versus 36 days, *p* = 0.011). However, in the multivariate analysis (MVA) adjusted for conditioning intensity, donor age, HCT-CI and DRI, and neutrophil and platelet engraftment had no significant differences between groups.

### 3.3. Graft-Versus-Host Disease

**Figure 1:**
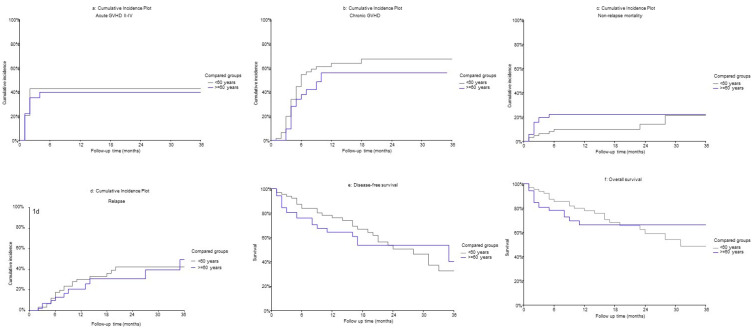
(**a**) Cumulative incidence of grade III–IV acute GVHD. (**b**) Cumulative incidence of chronic GVHD. (**c**) Cumulative incidence of non-relapse mortality. (**d**) Cumulative Incidence of Relapse. (**e**) Kaplan–Meier curve for disease-free survival. (**f**) Kaplan–Meier curve for overall survival. Blue line, ≥60 years. Grey line, <60 years.

At day 100, the cumulative incidence of grade II-IV acute GVHD was 42% in the younger group and 35% in the older group (SHR 0.93; 95% CI 0.49–1.74; *p* = 0.814) (Figure 1a). The cumulative incidence of grade III–IV acute GVHD was 8% in the younger group and 15% in the older group (SHR 2.25; 95% CI 0.57–8.82; *p* = 0.245). The cumulative incidence of chronic GVHD at 2 years was 67% in the younger group and 56% in the older group (SHR 0.70; 95% CI 0.41–1.20; *p* = 0.196) (Figure 1b). In the competing-risk regression analysis, while patient age was not significantly associated with acute or chronic GVHD (Table 2), higher HCT-CI was associated with an increased risk of chronic GVHD (SHR = 1.88, CI = 1.09–3.32, *p* = 0.022).

### 3.4. Non-Relapse Mortality and Relapse

At 2 years, the cumulative incidence of NRM in the younger patient group was 14%, and in the older patient group, it was 22% (univariate SHR 1.87; 95% CI 0.77–4.54; *p* = 0.167) (Figure 1c). In competing risk regression analysis, adjusting for donor age, HCT-CI, DRI, and conditioning intensity, the older group was at a significantly increased risk for NRM compared with the younger group (SHR = 4.05, CI = 1.43–11.47, *p* = 0.008). NRM was also significantly lower in patients receiving reduced intensity conditioning (SHR = 0.25, CI = 0.08–0.75, *p* = 0.014). Among the 15 NRM deaths in the older group, the causes of death were infection (n = 5), cardiopulmonary failure (n = 5), GVHD (n = 3), vaso-occlusive disease of the liver (n = 1), and mechanical injury with head trauma (n = 1).

At 2 years, the cumulative incidence of relapse was 42% for the younger group and 31% for the older group (univariate SHR 0.86; 95% CI 0.42–1.77, *p* = 0.690) (Figure 1d). Age was not significantly associated with relapse (*p* = 0.468) in competing-risk regression analysis (Table 3). High/very high DRI was associated with a significantly higher risk of relapse compared to low DRI (SHR = 2.68, CI = 1.12–6.37, *p* = 0.025).

### 3.5. Disease-Free and Overall Survival

The 2-year DFS for the younger and older group, respectively, was 51% and 53% (*p* = 0.72) (Figure 1e). In multivariate analysis, when adjusting for HCT-CI, DRI, conditioning intensity, and donor age, patient age was not correlated with DFS. DFS was significantly worse for patients with higher HCT-CI (HR = 1.87, CI = 1.04–3.34, *p* = 0.042). Similarly, compared to low DRI, DFS was significantly worse in patients with high/very high DRI (HR = 7.95, CI = 1.05–60.06, *p* = 0.002).

The 2-year OS for the younger group was 59%, and for the older group, it was 66% (*p* = 0.916) (Figure 1f). In the MVA, adjusting for HCT-CI, DRI and conditioning intensity, patient age was not associated with OS (*p* = 0.160). OS was significantly worse with the high HCT-CI ≥ 3 (HR = 1.98, CI = 1.03–3.84, *p* = 0.042) compared to low HCT-CI ≤ 3. There were no deaths in the low-DRI group, and therefore a comparison with intermediate or high/very high DRI was not possible.

### 3.6. Outcomes for Age ≥ 60 Years by Conditioning Regimen

Subgroup analysis was pursued to compare survival by conditioning regimen in the older population group. Comparing Flu/Bu (3500/5300) to Flu/Cy/TBI 200 cGy, the incidence of relapse was 11% vs. 41% (*p* = 0.156), NRM was 40% vs. 15% (*p* = 0.10), DFS was 53% vs. 49% (*p* = 0.83), and OS was 53% vs. 71% (*p* = 0.121), respectively.

### 3.7. Survival Outcomes for Age ≥ 70 Years

Fourteen patients in this study were over 70 years of age at the time of transplant. In this older group, the 2-year NRM was 8%. The 2-year cumulative incidence of relapse among this group was 32%. The 2-year DFS was 51%, and the 2-year OS was 76%.

## 4. Discussion

This study aims to evaluate the safety and efficacy of haplo PBSCT with PTCy in older patients who may be more sensitive to the higher incidence of GVHD that is associated with this graft source [8,9,10]. This study is of particular importance, as the results show that older patients had similar DFS and OS compared to the younger group, confirming that PBSCT with PTCy is an acceptable approach in older patients.

Prior studies of RIC-matched donor transplants in older patients have shown rates of long-term OS of 25–50% and NRM of ~10–30% [31,32,33]. Reports of haplo transplant with PTCy in older patients thus far have focused on marrow grafts, which are known to cause a lower risk of GVHD. Kasamon, et al. reported a single institution experience of 115 patients over 50 years of age who received nonmyeloablative haplo bone marrow transplants and found an NRM of 8%, DFS of 40%, and OS of 49% [13]. Further, when stratified by age 50–59 years versus 60–69 years versus 70–75 years, these outcomes were similar between groups. A subsequent study limited to patients above 70 years of age receiving nonmyeloablative haplo with PTCy and primarily marrow grafts also showed favorable NRM rates of 14%, and OS of 53% at 2 years [34]. Our study shows that haplo PBSCT with PTCy results in rates of NRM and OS that appear similar to these historically reported outcomes with matched donor or haplo BMT.

Many large retrospective studies have shown that patient age is not well correlated with post-transplant survival, but instead, outcomes are better predicted by baseline disease risk and comorbidities [35,36,37]. To assess if this conclusion is true following haplo PBSCT with PTCy, we compared outcomes in older patients to those of a younger patient population. Our MVA results did show a higher risk of NRM in the older patient group. However, the magnitude of this difference was not enough to influence a significant decrease in OS or DFS. Consequently, haplo PBSCT with PTCy in patients ≥60 years appears acceptably safe and effective. Further, in our subgroup of patients ≥70 years, outcomes also remained favorable with a 2-year OS estimate exceeding 70%. Rather than age, other factors, including the HCT-CI and DRI, were better indicators of outcomes, as these variables were associated with OS and DFS. Our results support that age should not be the primary consideration for predicting transplant outcomes after haplo PBSCT with PTCy, but instead a more rigorous assessment of comorbidities and disease risk are warranted.

While RIC regimens are often the only feasible option in older patients, specific regimens may yield variable outcomes [24]. In patients ≥60 years, we compared the two most commonly used conditioning regimens at our institution: Flu/Bu versus Flu/Cy/TBI. Our sample size was not large enough to detect significant differences in outcomes between these regimens. However, there was a trend toward improved OS with Flu/Cy/TBI attributable to a lower risk of NRM though higher risk of relapse. Further, while all patients in this study received a uniform dose of PTCy, there is increasing interest in reducing the dose of PTCy to mitigate chemotherapy-related toxicity in older and frail patients [38,39]. Thus, additional studies should explore the optimal conditioning and PTCy dosing for older patients receiving haplo PBSCT with PTCy.

Though we show that the outcomes of haplo PBSCT with PTCy in older patients are similar to those of young patients, our study does have notable limitations. In this retrospective study, the sample size was limited by the total number of patients treated at our institution. A post hoc power analysis shows that a two-sided log rank test with an overall sample size of 121 subjects provides 39% power at a 0.05 significance level to detect a hazard ratio of 0.60. Furthermore, in this retrospective cohort, the groups studied were heterogenous, particularly in regard to conditioning regimens. Due to the use of PBSC grafts as our institutional standard, we were unable to compare PBSCT to BMT in the older patient population. Nevertheless, we evaluated all consecutive eligible patients with the goal of avoiding selection bias. Additionally, to determine the optimal conditioning regimen for older patients receiving haplo PBSCT with PTCy, a larger cohort will be required and, ideally, would incorporate other regimens, such as melphalan-based regimens that have been adopted at some institutions [40].

## 5. Conclusions

The results of this study support the use of haplo PBSCT with PTCy as an appropriate transplant platform for older patients. Survival outcomes in this patient cohort appear similar to those observed in younger patients. Further, our results are comparable to historical outcomes in older patients with other RIC transplant platforms. Future studies should investigate the optimal conditioning regimen when using haplo PBSCT with PTCy in older patients. A comparison of haplo BMT versus haplo PBSCT in older patients is also warranted.

## Figures and Tables

**Table 1:** Baseline characteristics.

Variable		<60	≥60	Total	*p*-Value
N		66	55	121	
Median patient age (range)		42 (20–59)	66 (61–75)	58	
Gender	Male	40 (61%)	37 (67%)	77 (64%)	0.448
	Female	26 (39%)	18 (33%)	44 (36%)	
Median KPS		90 (IQR 10)	90 (IQR 10)	90 (IQR 10)	0.998
DRI	Low	5 (7%)	3 (5%)	8 (7%)	0.075
	Intermediate	42 (64%)	45 (82%)	87 (72%)	
	High/very high	19 (29%)	7 (13%)	26 (21%)	
HCT-CI	0–2	27 (41%)	24 (44%)	51 (42%)	0.763
	3+	39 (59%)	31(56%)	70 (58%)	
Conditioning regimen intensity	Myeloablative	55 (83%)	15 (27%)	70 (58%)	<0.001
	Reduced intensity	11 (17%)	40 (73%)	51 (42%)	
Disease	AML	29 (44%)	31 (56%)	60 (50%)	0.002
	MDS or MPN	12 (18%)	18 (33%)	30 (25%)	
	ALL	20 (30%)	1 (2%)	21 (17%)	
	Lymphoma	5 (8%)	5 (9%)	8 (8%)	
CMV recipient	Positive	46 (70%)	37 (69%)	83 (69%)	0.519
	Negative	20 (30%)	17 (31%)	37 (31%)	
Median donor age (range)		33 (IQR 21)	41 (IQR 13)	36 (IQR 17)	0.006

**Table 2:** Multivariate analysis for GVHD outcomes.

Multivariate Competing Risk Regression Analysis for GVHD Outcomes			
Acute GVHD II–IV			
**Covariate**	**Level**	**SHR (95% CI)**	***p*-Value**
Age	<60	1	
	≥60	0.92 (0.49–1.74)	0.814
**Acute GVHD III–IV**			
Age	<60	1	
	≥60	2.24 (0.57–8.82)	0.245
**Chronic GVHD**			
Age	<60	1	
	≥60	0.70 (0.41–1.20)	0.196
HCT-CI	0–2	1	
	≥3	1.88 (1.09–3.34)	**0.022**

**Table 3:** Multivariate competing risk analyses of survival outcomes.

Relapse			
Covariate	Level		*p*-Value
Age	<60	1	
	≥60	SHR 0.74 (95% CI 0.33–1.66)	0.468
Disease Risk Index	Low	1	
	Int	SHR 2.02 (95% CI 0.31–13.34)	0.731
	High/very high	SHR 2.68 (95% CI 1.12–6.37)	**0.025**
**NRM**			
Age	<60	1	
	≥60	SHR 4.05 (95% CI 1.43–11.47)	**0.008**
Conditioning	Myeloablative	1	
	Reduced intensity	SHR 0.25 (95% CI 0.08–0.75)	**0.014**
**DFS**			
Age	<60	1	
	≥60	HR 1.49 (95% CI 0.78–2.85)	0.230
HCT-CI	0–2	1	
	≥3	HR 1.87 (95% CI 1.04–3.34)	0.035
Disease Risk Index	Low	1	
	Int	HR 3.04 (95% CI 0.41–22.37)	0.281
	High/very high	HR 7.95 (95% CI 1.05–60.06)	**0.002**
**OS**			
Age	<60	1	
	≥60	HR 1.66 (95% CI 0.79–3.49)	0.182
HCT-CI	0–2	1	
	≥3	HR 1.98 (95% CI 1.03–3.84)	**0.042**

## Data Availability

The data that support the findings of this study are available from the corresponding author upon reasonable request.
